# Does the intensity of pain and disability affect health-related quality of life of older adults with back pain? Multilevel analysis between Brazil and Netherlands: a cross-sectional study of the BACE consortium

**DOI:** 10.1186/s12877-024-04803-x

**Published:** 2024-03-05

**Authors:** Adriana Estela de Oliveira Grigorini, Leani Souza Máximo Pereira, Bart Koes, Silvia Lanzioti Azevedo da Silva, Alessandro Chiarotto, Diogo Carvalho Felício, Amanda Aparecida Oliveira Leopoldino

**Affiliations:** 1 Postgraduate Program in Health Sciences, Faculdade Ciências Médicas de Minas Gerais, 275 Alameda Ezequiel Dias, 30130-110 Belo Horizonte, Minas Gerais Brazil; 2 Department of Physical Therapy, Graduate Program in Rehabilitation Science, Universidade Federal de Minas Gerais, Belo Horizonte, Minas Gerais Brazil; 3 Department of General Practice, Erasmus MC, University Medical Center, Rotterdam, The Netherlands; 4 Department of Collective Health, Faculty of Medicine, Universidade Federal de Juiz de Fora, Juiz de Fora, Brazil; 5 Department of Physical Therapy, Universidade Federal de Juiz de Fora, Juiz de Fora, Brazil

**Keywords:** Aged, Back pain, Quality of life, Disability, Multicenter study

## Abstract

**Background:**

The prognosis of back pain (BP) in the older adults is less favorable than in younger adults and progress to adverse outcomes and consequent worsening of health-related quality of life (HRQoL). The present study aimed to verify the association between BP intensity, disability and HRQoL in older adults residents in Brazil and Netherlands, and to evaluate whether the country of residence influences the associations.

**Methods:**

Data were collected from 602 Brazilian and 675 Dutch participants with a new episode of BP from the Back Complaints in Elders (BACE) consortium. For the present study, a cross section was used. Pain intensity and disability were assessed using the Numerical Rating Scale (NRS) and the Roland-Morris Disability Questionnaire (RMDQ), respectively. HRQoL was assessed using the Short Form Health Survey (SF-36) quality of life questionnaire. Age, sex, and education were descriptive variables. Pain intensity (NPS score) and country were the independent variables and quality of life assessed by each SF domain − 36 was the dependent variable. Analysis of models at the individual level was performed to verify the association between pain and disability, also HRQoL in Netherlands and Brazil in the total sample. The multilevel model was used to verify whether the older adults person’s country of residence influenced this relationship.

**Results:**

The average age of the participants was 67.00 (7.33) years. In the total sample, linear regression analysis adjusted for sex and age showed a significant association between BP intensity scores and HRQoL, for all domains. There was no association between disability and HRQoL. In the multilevel analysis, there was an association between BP intensity and HRQoL in all domains and an association between the country of residence and HRQoL, influencing the effect of pain, in all domains, except for the physical functioning.

**Conclusion:**

Socioeconomic and cultural aspects of different countries can affect the perception of the elderly about their HRQoL in the presence of BP. Pain and disability in Brazilian and Dutch older adults ones are experienced differently in relation to their HRQoL.

## Background

Back pain (BP) is a major musculoskeletal problem that causes functional limitations and suboptimal health-related quality of life (HRQoL) in older adults [1]. BP is a common symptom experienced by people of all ages, and its incidence and prevalence increase with age [2, 3]. The prevalence of BP in Brazilian older adults is around 33.6–68.3% [4], while in Dutch individuals the prevalence is around 36.4% for older adults over 65 years [5].

BP is a global health problem, being the main cause of Years Lived with Disability in 2016 and its burden is growing along with the growth and aging population resulting in greater dependence and vulnerability [6–8]. Increased incidence of disability and pain intensity in patients with BP is an important predictor of worsening HRQoL, impairing healthy life expectancy [9, 10]. According to the World Health Organization, HRQoL is “a broad concept affected in complex ways by a person’s physical health, psychological state, personal beliefs, social relationships and their relationship to salient features of their environment” [11].

Previous studies have shown that there are important differences in pain intensity, physical disability and HRQoL in different health services and in different racial subgroups, sex, weight, level of physical activity and patient age [8, 12–14]. However, few studies have evaluated the association between BP and country of residence, and it is reasonable to argue that pain severity and disability may be associated with social, cultural, economic and lifestyle differences in older adults people between high-income countries and middle/low-income countries may have different outcomes [15, 16]. In a recent study, it was observed that adverse physical HRQoL trajectories were more likely among individuals recently affected by economic, social or stressful life events, especially not undertaking paid work, with money problems, loneliness and illness of family/friends [17].

Thus, other factors beyond pathophysiological influences may explain inter-individual differences in pain intensity, disability, HRQoL in the remarkably heterogeneous population of older adults people with BP [18]. Therefore, the objective of the present study was to verify the association between BP intensity and disability with HRQoL in older adults residents in Brazil and Netherlands, and to evaluate whether the country of residence influences the associations.

## Methods

### Study design

This was an observational, cross-sectional, ancillary study of the BACE, an international multicenter cohort study [19]. The paper followed the Strengthening of the Reporting on Observational studies in Epidemiology (STROBE) guidelines for writing observational studies [20].

### Ethical consideration

The BACE-Brazil project was approved by the Research Ethics Committee in 2011 (ETIC 0100.0.203.000–11). The BACE Netherlands project was approved by the Medical Ethics Committee of Erasmus MC (NL 24829.078.08). All older people manually signed the informed consent form.

### Sample

The selection of the BACE Brazil sample was carried out by health professionals who referred individuals with BP complaints for contact with the BACE team. A total of 3711 participants were screened. Exclusion criteria were: age less than 55 years (*n* = 376); episode of non-acute pain (*n* = 1803); no new BP episode (*n* = 270); severe visual or hearing problems (*n* = 12); severe motor dysfunctions (*n* = 5); severe cognitive dysfunctions (*n* = 7) (Mini-Mental State, Folstein et al., 1975) [21]; two or more reasons (*n* = 123); not consenting to participate (*n* = 264) and other reasons (*n* = 249). The final sample of BACE Brazil sample added up to 602 individuals.

The selection of the BACE Netherlands sample was carried out by screening all patients with BP, aged > 55 years, in consultation with the general practitioner. Patients invited to participate (*n* = 1402), directly during the consultation (*n* = 141) and in writing after the consultation (*n* = 1261). After this selection, the following ones were excluded: patients unwilling to participate (*n* = 291); patients who did not meet the inclusion criteria (*n* = 118); no new episodes of back pain (*n* = 77); language problem (*n* = 12); cognitive disorder (*n* = 5), (Mini-Mental State, Brucki et al. 2003) [22]; no back pain consultation (*n* = 24); did not respond (*n* = 318). The final sample: Inclusion (*n* = 675) via A: directly at the appointment (*n* = 105) and via B: In writing after the appointment (*n* = 570).

In this current study, in the BACE Netherlands sample, seven individuals were excluded because HRQoL data was unavailable.

### Measurement instruments

The descriptive variables of the present study were: age, sex, and education (in years). The dependent variable is composed of the physical and mental health of HRQoL, and the independent variables were pain intensity and disability.

### Health-related quality of life

HRQoL was assessed using the Short Form Health Survey (SF-36) questionnaire. The SF-36 has good construct validity, high internal consistency (with Cronbach’s alphas ranging from 0.85 to 0.94 for individual subscales), and high test-retest reliability to measure HRQoL in older adults individuals [23]. It contains eight subscales: physical functioning subscale (10 items), role physical subscale (4 items), bodily pain (2 items), general health status (5 items), vitality (4 items), social functioning (2 items), role emotional (3 items) and mental health (5 items) categorized into two scores: the Physical Component Score which includes physical functioning, role limitations due to physical health problems, bodily pain and general health, and the Mental Component Score which includes vitality, role limitations due to emotional problems, social functioning and mental health. The scores are then transformed into a scale ranging from zero (reflecting poor HRQoL) to 100 (reflecting excellent HRQoL) [23–25]. For the present study, the eight subscales of the SF-36 were used.

#### Pain

Pain intensity was measured using the Numerical Rating Scale (NRS). It is a simple instrument with high test-retest and reproducibility to measure pain intensity in older adults individuals [26]. BP can be classified as none (score 0), mild (1–3), moderate (4–6) and severe (7–10) [27].

#### Disability

Disability was assessed using the 24-item Roland-Morris Disability Questionnaire (RMDQ), which reflects functional changes due to BP [28]. It has a standardized scoring system and the higher the score, the greater the degree of disability of the individual [29]. The questionnaire is quick and easy to manage, takes an average of five minutes and can be easily scored. This instrument consists of 24 questions that address the functional limitations resulting from BP. The answer to all questions is dichotomous: “yes” (1 point) or “no” (0), and the total score is the sum of positive answers, ranging from zero to 24 points. Values greater than 14 points indicate physical disability [30]. Baseline scores less than 4 RMDQ points indicate low levels of disability and baseline scores greater than 20 RMDQ indicate high levels of disability [30]. The same RMDQ was used in the studies in Brazil and the Netherlands. The questionnaires had sufficient construct validity and responsiveness. Internal consistency Cronbach α of 0.89 and test-retest reliability (ICC 2,1 of 0.85) [28, 32, 33].

#### Covariates

The sociodemographic variables for characterizing the sample that were selected for this study are: age, sex, and education. Previous research has shown that BP increases with age, and that female sex is significantly associated with BP [15]. Other studies of diverse global populations have also reported inverse socioeconomic gradients between the prevalence and intensity of BP, along with education and wealth, which suggest that long-term effects of social and economic adversities may influence health conditions in later life [15, 34].

### Statistical analysis

Statistical analysis was performed in three steps: sample description; models at the individual level, to verify the association between pain and disability and HRQoL in the Netherlands, Brazil and in total sample; multilevel model to verify whether the older adults place of residence influenced the relationship between disability, pain and quality of life. The place of residence is an independent variable measured at a more external and collective level, which is why the use of the multilevel model. In step 1, the sample was presented using descriptive statistics. In step 2, an analysis was carried out with the total sample, and another stratified by country, Brazil or Netherlands. The models were adjusted for sex and age, to verify the association between pain/disability for each of the eight quality of life domains. In step 3, multilevel analysis was performed, only for the independent variable pain, which was associated with HRQoL in the individual models. The first part of this model is the evaluation of the assumptions of the multilevel model, theoretically established: (1) establishment of the level of the model variables: level 2 (group): country (two options) and level 1 (individual): older adults; (2) independent variables: pain intensity (NRS score), and country. Dependent variable: quality of life assessed by each SF − 36 domain– measured at the same level as the closest independent variable (pain). Step 3 was carried out just for pain due the step 2 results The final sample was composed by 1270 seniors (seven seniors excluded for not having SF-36 value). All variables were analyzed as numerical. Descriptive variables of the sample were: age, sex, education; (3) relationship between variables (hypothesis): pain is associated with HRQoL in the older adults, and the country where the older adults person lives influences this association; (4) interaction between levels (hypothesis): country influences the relationship between pain and quality of life, with a moderating effect of country on the relationship between pain and quality of life. After the theoretical stages, the relationship between the independent variable at the individual level (pain) between the groups of the most external variables (country) were observed, by the values (1) measure of agreement (RGW = 632.5); (2) deviance = 2.261 and (3) reliability (F Test *p* = 0.001). All assumptions were fulfilled, enabling the multilevel analysis to be carried out. This analysis served for all HRQoL domains examined, as the independent variables are the same.

The analyzes were performed using the R software version 4.1.0 using “Multilevel” packages, significance level of 0.05 was considered.

## Results

The evaluated sample consisted of 1270 participants who were categorized into two groups: Brazilians (*n* = 602, 67.70 ± 7.00 years) and Dutch (*n* = 668, 66.40 ± 7.57 years). As for sociodemographic characteristics, 908 (71.5%) of all participants were female and 362 (28.5%) were male, with a mean age of 67.00 (± 7.33). Brazilian older adults had lower educational levels and worse scores for disability (RMDQ) and pain intensity (NPS) compared to Dutch older adults. The sample characteristics at baseline are presented in Table 1.

**Table 1:** General characteristic of participants at baseline (*N* = 1270)

Variable	Total Sample (*n* = 1270)	Brazil (*n* = 602)	Netherlands (*n* = 668)
Age (years)	67.00 (± 7.33)	67.67 (± 7.00)	66.40 (± 7.57)
Sex			
Male	362 (28.5%)	91 (15.1%)	271 (40.6%)
Female	908 (71.5%)	511 (84.9%)	397 (59.4%)
Schooling			
1	44 (3.4%)	41 (6.8%)	3 (0.4%)
2	307 (24.1%)	219 (36.3%)	88 (13.1%)
3	303 (23.8%)	116 (19.2%)	187 (28.0%)
4	295 (23.2%)	110 (18.2%)	185 (27.7%)
5	48 (3.7%)	23 (3.8%)	25 (3.7%)
6	116 (9.1%)	51 (8.4%)	65 (9.7%)
7	133 (10.4%)	41 (7.3%)	92 (13.7%)
8	22 (2.3%)	0	22 (3.7%)
NRS	6.12 (± 2.82)	7.17 (± 2.59)	5.16 (± 2.68)
RMDQ SF-36	8.17 (± 7,24)	9.99 (± 7.81)	6.52 (± 6.24)
Physical role functioning	20.35 (± 5.25)	1863 (± 4,78)	2191 (± 5.17)
Physical functiong	5.35 (± 1.62)	5.13 (± 1.46)	555 (± 1.73)
Bodily pain	6.22 (± 2.07)	6.00 (± 2.12)	6.42 (± 200)
General health perceptions	16.95 (± 4.18)	16.88 (± 4.32)	17.01 (± 4.06)
Vitality	14.95 (± 4.57)	1390 (± 4.86)	15.89 (± 4.07)
Social role functioning	7.36 (± 2.16)	6.84 (± 2,0.28)	7.83 (± 1.94)
Emotional role functioning	4.83 (± 1.37)	4.42 (± 1.37)	5.20 (± 1.27)
Mental health	21.77 (± 5.70)	19.84 (± 6.18)	23.51 (± 4.57)

N, number of participants; NRS, numeric rating scale; RMDQ, Roland Morris disability questionnaire; SF-36, 36-item Short Form Health Survey. Schooling 1 = 1 year; 2 = 2 years; 3 = 3 years; 4 = 4 years; 5 = 5 years; 6 = 6 years; 7 = 7 years; 8 = 8 or more years of schooling

Table 2 presents the results of the association between BP and each of the HRQoL domains of the older adults participants in both countries. It is observed that Dutch older adults had a greater impact of pain on their HRQoL assessed by the SF-36 (Table 2).

**Table 2:** Association between pain intensity and health-related quality of life measured by SF–36 in Brazilian and Dutch older adults

Outcome SF-36	Total Sample			Brazil			Netherlands		
	Beta 0	Beta 1	CI95%	Beta 0	Beta 1	CI95%	Beta 0	Beta 1	CI95%
Physical role functioning	25.76	-0.88	-0.97- -0.79	23.33	-0.65	-0.79- -0.51	26.42	-0.86	-1,00- -0.73
Physical functioning	6.50	-0.18	-0,21- -0,15	6.20	-0.14	-0.19- -0.10	6.66	-0.21	-0.26- -0.16
Bodily pain	8.28	-0.33	-0.37- -0.29	8.21	-0.30	-0.36- -0.24	8.46	-0.39	-0.44- -0.34
General health perceptions	19.01	-0.33	-0.41- -0.25	19.10	-0.30	-0.44- -0.17	19.24	-0.43	-0.54- -0.32
Vitality	17.75	-0.45	-0.54- -0.37	15.87	-0.27	-0.42- -0.12	18.31	-0.46	-0.57- -0.35
Social role functioning	8.77	-0.22	-0.26- -0.18	8.19	-0.18	-0.25- -0.11	8.84	-0.19	-0.24- -0.14
Emotional role functioning	5.61	-0.12	-0.15- -0.10	4.97	-0.07	-0.11- -0.03	5.72	-0.09	-0.13- -0.06
Mental health	25.07	-0.53	-0.64- -0.43	22.28	-0.34	-0.53- -0.51	25.37	-0.35	-0.47- -0.22

SF-36, 36-item Short Form Health Survey, Beta 0: Intercept of the model; Beta 1: coefficient of independent variable. Models adjusted by sex, age and education

After analyzing the data with the proposed models, it was found that there was no significant association between disability and all domains evaluated in the questionnaire that assesses the SF-36 questionnaire of the older adults participants in both countries (Table 3).

**Table 3:** Association between disability (RMDQ) and health-related quality of life measured by SF–36 of Brazilian and Dutch older adults

Outcome SF-36	Total Sample			Brazil			Netherlands		
	Beta 0	Beta 1	CI95%	Beta 0	Beta 1	CI95%	Beta 0	Beta 1	CI95%
Physical role functioning	20.83	-0.05	-0.09- -0.01	18.70	-0.006	-0.05- 0.04	21.91	-0000	-0.06- 0,06
Physical functioning	5.32	0.003	-0.01- 0,01	5.09	0004	-0.01- 0,01	5.41	0.020	-0.01- 0.04
Bodily pain	6.25	-0.003	-0.01- 0.02	5.97	0.003	-0.01- 0.02	6.40	0.003	-0.02- 0.02
General health perceptions	16.90	0.006	-0.02- 0.03	16.80	0.008	-0.03- 0,05	16.95	0.009	-0.04- 0.05
Vitality	15.11	-0.019	-0.05- 0.01	13.86	0.003	-0.04- 0,05	15.71	0.028	-0.02- 0.07
Social role functioning	7.42	-0,0.007	-0.02- 0,01	6.77	0.007	-0.01- 0,03	7.74	0.013	-0.01- 0.03
Emotional role functioning	4.87	-0.005	-0.01- 0,01	4.37	0.005	-0,01- 0,02	5.12	0.013	-0.01- 0,02
Mental health	22.08	-0.037	-0.08- 0,01	19.61	0.022	-0.04- 0,08	23.34	0.027	-0.02- 0.08

SF-36, 36-item short form Short Form Health Survey. Models adjusted by sex, age and education

When performing the multilevel analysis with a sample of older adults people from both countries, it was found that the country of residence influenced the association between pain and HRQoL in the following SF-36 domains: physical role functioning, vitality, social function, role emotional, and mental health (Table 4).

**Table 4:** Multilevel model

Variable Dependent	Intercept	Fixed Effect - Pain			Random Effect - Country			Residual
SF-36		Estimate	Standard Error	p	Estimate	Standard Error	p	
Physical role functioning	30.401	-0.770	0.048	< 0.001	-0.868	0.135	< 0.001	4.55 (± 0.48)
Physical functioning	6.604	-0.185	0.016	< 0.001	-0.019	0.045	0.678	1.53 (± 0.16)
Bodily pain	7.502	-0.354	0.019	< 0.001	0.146	0.055	0.007	1.84 (± 0.19)
General health perceptions	17.367	-0.374	0.043	< 0.001	0.308	0.120	0.010	4.05 (± 0.42)
Vitality	21.055	-0.378	0.046	< 0.001	-0.618	0.130	< 0.001	4.35 (± 0.46)
Social role functioning	10.394	-0.190	0.021	< 0.001	-0.303	0.061	< 0.001	2.05 (± 0.21)
Emotional role functioning	7.246	-0.088	0.013	< 0.001	-0.304	0.038	< 0.001	1.29 (± 0.13)
Mental health	33.051	-0.347	0.056	< 0.001	-1.492	0.158	< 0.001	5.30 (± 0.56)

SF-36, 36-item short form Short Form Health Survey

## Discussion

The results showed that the intensity of BP and disability have a different impact on the HRQoL of older adults residents in Brazil and in the Netherlands. It was also observed that the country of residence influences the impact of pain on the HRQoL. Both countries have different cultures and socioeconomic levels, which may explain the difference in the impact of HRQoL in both countries.

### Back pain and HRQoL

Previous evidence indicate that BP have a high burden of disability, increasing with age, with a peak around 80 years of age [34]. HRQoL is impacted by chronic BP in different domains of life, such as physical and mental well-being, social relationships, and functional capacity [23]. In a recent study, greater pain intensity was associated with the perception of BP as a greater threat and lower HRQoL [35]; the more pain participants experienced, the worse their HRQoL [36].. A comparison between individuals who reported BP and matched pain-free controls indicated that older adults people with BP reported significantly impaired HRQoL scores and a significantly lower self-assessment of general health [37]. In the present study, participants from both countries had an impact of the intensity of BP on their HRQoL, but the intensity of pain reflected on HRQoL was lower in Brazilian older adults.

### HRQoL and country of residence

When analyzing the results of the BACE-Netherlands sample, it was possible to observe that BP intensity had a greater impact on the HRQoL (physical role functioning, vitality, social function, role emotional, and mental health) from Dutch individuals. On the other hand, in BACE-Brazil sample, Brazilians had a worse perception of pain and general health status, as assessed by the SF-36. A recent study concluded that a lower socioeconomic status was associated with poor physical HRQoL in the older adults [17]. In another study, participants’ pain experiences were profoundly informed by a combination of experiences that included discrimination, stigma, dismissal, and social and economic disadvantage, as well as trauma experiences in marginalized communities [38]. When analyzing data provided by the United Nations on the Human Development Index (HDI) from Brazil and Netherlands in 2022, Brazil fell from 86th to 87th (0.754) in the human development ranking, while Netherlands ranked 7th (0.890) in the world ranking [39]. Both countries have different cultures and values, divergent levels of individual and national wealth, slightly different life expectancies [40], which may have contributed to different perceptions and importance of HRQoL.

While life expectancy at birth in Netherlands is 82 years [41], Brazil is 76.3 years [42]. The total gross domestic product of both countries is practically the same, but population of Brazil is almost 13 times greater population than Netherlands [43]. In Brazil, Multidimensional Poverty Index is still calculated, which identifies how people are being left behind in three main dimensions: health, education, and standard of living, comprising ten indicators. This indicator is not calculated in Netherlands [39].

### Back pain, HRQoL and resilience

According to our outcomes, when dealing with BP, it seems necessary to include psychological, social, and cultural aspects in addition to biological ones. One aspect which can support the explanation of our results on why HRQoL was more impacted in Dutch older adults people is resilience [6]. The resilience which in old age translates into a process of positive adaptation to adversity, trauma, threats, or significant stress and encompasses content such as: feeling of being competent even accepting help from others, being active, looking positively at life and living connected to the present [6, 43]. The nature of adversity experienced over a lifetime can differ between regions of the world, with people in poor countries generally experiencing greater and more severe adversity [31]. Complex interactions between socioeconomic realities, social connections, and psychological assets also shape resilience, an attribute that appears to compensate for adversity and enhance physical, also mental health across lifespan [40]. Resilience can, therefore, be a fundamental resource that facilitates adaptation to chronic low back pain [44]. It can increase self-assessment of health [44]. Worse socioeconomic conditions, low educational level, worst HDI, higher poverty level, larger population for the same GDP, are characteristics of the country Brazil, which can confer greater resilience to the Brazilian population, that even with higher pain levels, the impact of this pain in quality of life was lower than in people from developed countries who experienced lower levels of pain.

### HRQoL and disability

Otherwise, it was observed that there was no association between disability and.

HRQoL, which does not corroborate with previous evidence. In a previous study, it was observed that the inability to perform activities of daily living in the older adults decreases their quality of life [45]. However, in a recent study by our group, with equal sample as the current study, using statistical analysis of latent class, four trajectories for disability were identified [46]. Disability trajectories varied over the course of a year, and could present with complete, incomplete, persistent mild to moderate recovery, and severe disability. It is important to consider that the trajectories of pain and disability change over time and depend on biopsychosocial and clinical factors, extending life habits to be carried out at the time of the assessment [46]. Another explanation is that disability attributed to BP varies substantially across countries and it is influenced by social norms, local health approaches, and legislation [3]. A Japanese study demonstrated that BP is associated with functional disability, and such association is observed to be stronger in a dose-dependent manner, which includes the intensity, frequency, and duration of BP [47]. In the present study the sample was considered as a whole, which may have masked possible effects in subgroups. In the case of pain and HRQoL, it relates, among other aspects, to subjective and perceptual issues, which may justify the results between pain and quality of life. Disability, on the other hand, was evaluated in a more objective way, questioning the difficulties in carrying out specific and objective day-to-day activities.

### Clinical implication

Our findings highlight the need for a different perspective when approaching the older adults with BP. In addition to the impact of biological issues, an analysis of the socioeconomic, cultural, psychological and lifestyle issues of the older adults is also necessary, allowing a greater chance of resolving the BP. In addition, implementing public policies with grassroots approaches are needed to emphasize economic autonomy, social support networks and coping skills in order to support the physical well-being of the older adults [1].

### Strengths and limitations

Our study has several strengths: (a) the data were collected by a team of well-trained researchers; (b) a standardized approach was used to define all study variables; (c) international comparison between databases; (d) the use of multilevel analysis.

The limitations of this study refer to: (a) the convenience sampling which restricted the characteristics of the participants, limiting the external validity of the study; (b) the BP is self-reported, but this type of assessment is common due to the subjectivity of BP and it is well accepted in epidemiological studies; (c) the analysis adjusts for age and sex, but other relevant factors influencing HRQoL, such as comorbidities or psychological well-being, are not extensively addressed.

## Conclusion

This study showed the socioeconomic and cultural aspects from different countries which can impact the perception of the older adults about their HRQoL in the presence of BP. Pain and disability in Brazilian and Dutch older adults are experienced differently in relation to their HRQoL. Resilience can be a mechanism for coping with the adversities experienced by the older adults, providing a better assessment of health, and public health policies can be created to reduce social and economic disparities.

## Abbreviations

BACE: Back Complaints in Elders
BP: Back pain
GDP: Gross domestic product
HDI: Human Development Index
HRQoL: Health-related quality of life
NRS: Numerical Rating Scale
RMDQ: Roland-Morris Disability Questionnaire
SF-36: Short Form Health Survey
